# Knowledge Connects Our Hearts and Lands: A Qualitative Research Study on Stewarding Indigenous Traditional Ecological Knowledges for Community Well-Being

**DOI:** 10.3390/ijerph22101573

**Published:** 2025-10-15

**Authors:** Danya Carroll, Desiree J. Edwards, Ramon Riley, Nicole Redvers

**Affiliations:** 1 Schulich School of Medicine and Dentistry, Western University, London, ON N6G 2M1, Canada; dcarro4@uwo.ca; 2 College of Social and Behavioral Sciences, University of Arizona, Tucson, AZ 85721, USA; desireejedwards@arizona.edu; 3 CD Broken Arrow CC, Whiteriver, AZ 85941, USA; rileyhali41@gmail.com; 4 Arctic Indigenous Wellness Foundation, Yellowknife, NT X1A 0C6, Canada

**Keywords:** Indigenous Peoples, traditional ecological knowledge, Indigenous knowledge, Indigenous knowledge systems, qualitative research, data stewardship, Indigenous data

## Abstract

Indigenous Peoples have developed and stewarded complex knowledge systems that have contributed to thriving societies. With continued threats to Indigenous lifeways, there is increasing need to further protect traditional ecological knowledges (TEK). We carried out a qualitative study to explore Indigenous community perspectives on stewarding and protecting TEK while identifying gaps in community-level protections of TEK. We conducted ten semi-structured interviews in December 2024 and one focus group in January 2025 with Indigenous Peoples in the southwestern United States. Reflexive thematic analysis through open coding was carried out using qualitative software. Six overarching themes were characterized in the interviews, which overlapped with findings from the focus group, including the following: (1) Historical and current barriers impact the sharing of TEK; (2) Preserving our language is necessary for intergenerational transmission of our TEK; (3) Our TEK reveals changes to our Lands; (4) Protecting our Lands and medicines is vital to our health; (5) We must take the time to learn our TEK for future generations; and (6) We need to protect our TEK. Our research highlights the importance of supporting Indigenous communities’ capacities to protect their TEK for personal, community, and environmental well-being.

## 1. Introduction

Indigenous knowledges, including traditional ecological knowledges (TEK), are recognized as long-standing and context-specific holistic knowledge systems that have been stewarded by Indigenous communities for millennia [1,2]. Despite numerous and ongoing attempts to eradicate Indigenous knowledges such as TEK, Indigenous Peoples have effectively stewarded their Lands in many places around the world [3]. This stewardship has been in the face of Indigenous land rights violations and forced removal from traditional territories [4,5]. Nevertheless, Indigenous Peoples have still steadfastly stewarded the majority of the remaining global biodiversity [6]. Through kinship-driven processes (i.e., Nature viewed as kin), Indigenous Peoples, including those on Turtle Island (i.e., North America), continue to promote the importance of caring for their Lands through the active use of their knowledge systems [7]. Indigenous TEK and worldviews have also provided a valuable blueprint for fostering overall community health and well-being [8]. There is widespread recognition among Indigenous communities that healthy Indigenous Lands are necessary for healthy communities [9,10]. Therefore, through applications of TEK, caring for and protecting Lands, waters, traditional medicines, and non-human relatives is foundational to Indigenous Nations.

Indigenous Lands and waterways continue to be impacted by various factors, including climate change and resource extraction. In the southwestern United States (USA), Tribal communities are impacted by climate change in various ways, including prolonged droughts, decreasing water supply, and soil quality [11]. Climate change is concerning for many southwestern Tribal Nations as it continues to challenge relationships with culturally significant landscapes (e.g., Forests) and ecosystems [12]. The impacts of climate change threaten the TEK that comes from the Lands and the intergenerational transmission of TEK in these communities. Additionally, culturally significant and sacred sites that possess both cultural and ecological value continue to be subjected to desecration and destruction by industries, governments, and other corporations [13,14], leading to decreased access to important land bases for Indigenous Peoples. The continued assaults on culturally significant land bases can detrimentally disrupt access to Indigenous TEK and spiritual connections with these spaces for current and future generations. The continued and ongoing infringement of Indigenous Peoples’ rights to access and steward their Lands and traditional medicines will have major impacts on their ability to engage in the intergenerational transfer of TEK. Globally, the stewardship role of Indigenous Peoples is imperative for the sustainability and care of the planet [15,16].

TEK have historically and contemporarily been passed on intergenerationally within Indigenous communities—with particular leadership in this regard from Elders. Indigenous Elders are highly revered and respected in Indigenous communities, as they are seen as key knowledge keepers [17] and transmitters of TEK. Nevertheless, colonization and the Euro-Western-centric worldview that was forced on many Indigenous Nations often minimized the roles of Elders [17] and therefore disrupted the transmission of TEK. Colonization also brought with it Euro-Western-centric education systems where Indigenous knowledge systems and Indigenous worldviews were often outlawed or forcibly suppressed (e.g., boarding or residential schools in the USA and Canada), leading to the experience of historical trauma [18,19,20]. Colonization’s cumulative impacts have robbed subsequent generations of TEK-related teachings that often come from Indigenous Elders. Nevertheless, despite colonization and its continued effects, many Indigenous communities have remained resilient and have made strong efforts to ensure Elders and their knowledge are valued and their knowledge is passed on. The distinct knowledge and wisdom of Indigenous Elders is crucial to preserving and platforming TEK as being essential to the health and well-being of current and future generations [21].

TEK have most often been passed on orally and supplemented by direct lived experience on the ground in Indigenous communities. There is increasing recognition, however, of the need to secure and protect TEK-related materials in community-based repositories that promote overall Indigenous data sovereignty. Indigenous data sovereignty affirms that Indigenous Peoples have rights to own, collect, and govern the application of their data [22]. Many Indigenous communities are on the front lines of climate-induced impacts, including changing landscapes (e.g., erosion) and extreme weather. For example, coastal Indigenous communities in Alaska are facing more frequent destructive storms, which highlights the urgent need to secure their knowledges in accessible digital archives [23]. Many Alaska Native villages have had to relocate or plan to relocate due to severe climate-related flooding and erosion [24]. Community-based repositories vary but can be a physical and/or digital library that is responsive to the community’s needs, including prioritizing the secure storage of TEK-related materials before they are lost [25]. Community-based repositories are localized and managed and owned by an Indigenous community that seeks to establish and center self-determined approaches to managing their cultural information and proprietary traditional knowledge [25]. For example, the Rematriation Project creates capacity for Inuit communities to steward and protect their digital archives [24]. The Sípnuuk repository, developed by the Karuk Tribe, also ensures Indigenous data sovereignty is honored in the work [25].

A scoping review by Carroll et al. [26] identified and highlighted gaps in how Indigenous communities develop and strengthen their health and well-being knowledge repositories. These included a lack of Indigenous-led community-based accessible repository models that could be replicated in similar settings. These gaps highlighted the need for more resources to support Indigenous communities on the ground through this repository development or stewardship process. Consequently, there is a substantial need to support Indigenous communities’ capacity to develop and/or strengthen infrastructure to protect their knowledges, including TEK. Direct community input is required to further identify the needs of Indigenous stakeholders in repository development processes and overall TEK data sovereignty. Given the importance of listening to Indigenous community voices, we aimed to explore, through this qualitative study, Indigenous perspectives on how Indigenous communities steward and protect their TEK-related knowledges. We also wanted to further explore gaps in community-level efforts and capacity to protect TEK-related materials.

### Positionality

It is becoming increasingly recognized that research conducted with Indigenous Peoples, especially that related to Indigenous knowledges and epistemologies, must recognize and declare the positionalities of the author team [27,28,29]. Our study is an Indigenous-led research study that aligns with the tenet “Nothing about Indigenous Peoples, without Indigenous Peoples” [30]. We describe below our positionality as authors in this Indigenous research study that was conducted by, for, and with an Indigenous community [31]. The first author (DC) is an Indigenous community scholar from the Diné and White Mountain Apache Tribal Nations in the USA. The second author (DJE) is a White Mountain Apache geographic information systems (GIS) forestry professional from the USA. The third author (RR) is a White Mountain Apache Elder from the USA with vast experience in traditional knowledge protection and repatriation. The last author (NR) is an Indigenous community and planetary health scholar and a member of the Deninu K’ue First Nation in Canada.

## 2. Materials and Methods

### 2.1. Research Questions

This research study was carried out by an all-Indigenous research team, including three research team members that are from the community where the research was implemented. Research questions for this localized research study were: (1) How do Indigenous communities steward and protect their TEK? (2) What are the gaps in community-level protection of TEK? and (3) What protocols need to be strengthened for Indigenous TEK stewardship?

### 2.2. Overall Study Design

This qualitative research study was developed and led by an Indigenous team consisting of Indigenous scholars and an Indigenous Elder. Our team grounded this research study in an Indigenous conceptual framework that honored the local community [32]. Kovach describes the core foundations of this framework as integrating understandings of “Indigenous epistemology, Indigenous ethics, Indigenous community (including land and place), and the self” [33]. Indigenous epistemology encompasses beliefs and origins of knowledge, including whom it involves [33]. Indigenous ethics emphasize the role of researchers, including learning and committing to ethical protocols for Indigenous research conducted with communities [33]. All of these components were essential to our Indigenous conceptual framework while making space for an overarching decolonial approach [32]. This framework aligned with the local community’s epistemology and ethics, including prioritizing relationality, lived experience, and reciprocity.

Prior to beginning the research, it was vital for us to meet with local Indigenous Elders, including those on the local community cultural advisory committee, to discuss our research plan. Inclusion of Elder perspectives was vital to ensuring a culturally responsive approach that was respectful of local cultural protocols. For this study planning, we also drew upon the Storywork principles described by Archibald [34] and advanced by Galla and Goodwill [35]. Storywork emphasizes the significance of the spiritual, emotional, physical, and intellectual components in acquiring knowledge [34]. The Storywork principles also promote deep listening that is grounded in bringing together the heart and mind [35]. Archibald also expresses that “to learn the highest degree of cultural knowledge, one could go to an Elder or someone not yet an Elder who understands and who lives the ‘good’ cultural traditions [34].” As Elders often share holistically about topics of inquiry, discussions around research topics may appear to be broad to a Euro-Western audience but are fundamentally connected and interrelated to the topic at hand through Storywork. Given this, Storywork allows flexibility to Elders and knowledge holders in how they share information.

Through this research study we therefore aimed to honor the stories and knowledges shared by the participants through semi-structured interviews as well as a focus group. We used both data collection methods in order to honor multiple perspectives and approaches, as well as to enhance triangulation and to provide ample opportunities for “deep listening” and broader flexibility for sharing in a group. Triangulation can enhance understanding of study phenomena, including deeper understanding of knowledge [36]. This is consistent with Indigenous forms of triangulation that promote relational, culturally responsive approaches and sharing between participants and researchers [4]. The research study received ethics approval from the Western University Non-Medical Research Ethics Board (#125143), the local cultural advisory committee, and the Tribal Health Board and Tribal Council. Standards for reporting qualitative research (SRQR) were followed for this work [37].

### 2.3. Setting

This research study was conducted in a southwestern Indigenous Tribal Nation in the USA. The Tribal Nation consists of around 12,000 Tribal members, of which over 2500 reside on a reservation. The Tribal Nation continues to steward a large land base, including Forests, as it has for millennia. Community members continue to foster culturally rich environments, including intergenerational learning opportunities rooted in Land, ceremony, language, and culture. The community is highly relational and comprised of matriarchal clanship structures and still uses oral tradition to share knowledge, including TEK. Like many other Tribal Nations, the community experiences continued impacts of colonialization, including collective trauma. There are also health concerns that impact the population, including chronic diseases (e.g., diabetes, cancer) and mental health concerns. With a fairly large portion of the population being younger, there is a significant need to promote cultural continuity and overall community health and well-being. In respect of confidentiality and at the request of the Tribal Nation, we will not explicitly use their Tribal name or provide additional identifying information in this article.

### 2.4. Recruitment and Consent

After consulting with local knowledge holders, the researchers introduced the project to the local Tribal Health Board and a local cultural advisory committee consisting of Elders. Once the project was approved by the local Tribal government, an advertisement was placed in the local Tribal newspaper to support recruitment efforts. Community members were eligible to participate if they were Indigenous and over 18 years of age. Two locally based research team members using local community knowledge also identified potential participants with community-recognized experience and knowledge in cultural preservation, biodiversity stewardship, and environmental protection. Potential participants for the interviews and focus group were contacted by the research team members and provided information on the study. Research team members then scheduled interviews or the focus group with potential participants. A research team member fluent in the local Indigenous language translated the consent into the local Indigenous language. Potential participants were consented before the interviews or focus group in English and/or the local Indigenous language if preferred. The team met the interview participants at their preferred location (i.e., home or a central community location). The focus group took place in a community meeting room to ensure a quiet location. An honorarium was provided for each participant to compensate them for sharing their time and knowledge with our research team as per common protocol in Indigenous-based research. No participants withdrew or declined participation in the research study.

### 2.5. Interview and Focus Group Data Collection

Semi-structured interviews were conducted by two female Indigenous research team members in December 2024. All the participants were fluent in their Indigenous language as well as English. The interviews were carried out in either the local Indigenous language (*n* = 3) or in English (*n* = 7), with the language preference determined by each participant. The interviews were audio recorded and lasted between 30 and 75 min. The Indigenous research team co-developed the semi-structured interview guide with guidance from the local Tribal Health Board and cultural advisory committee. The interview guide (see Supplementary File S1) included questions on community stewardship around TEK, the connection between Land and TEK, and ways to protect TEK, including overcoming challenges to its protection, as well as how TEK is stored in the community.

A focus group was also conducted by two female Indigenous research team members. The focus group was carried out in January 2025 in the local Indigenous language with four participants. The focus group lasted for 84 min. The focus group allowed us to explore our research questions at the group level (see study design section). The focus group guide questions (see Supplementary File S2) were similar to the interview questions. Focus group questions addressed connection to the Land, how TEK is protected in the community, what the factors are (including Land) affecting the sharing of TEK, how TEK is stored/protected in the community, and what the needs are for future protection of TEK in the community.

### 2.6. Data Analysis

The interviews and focus group were transcribed verbatim using the NVivo Transcription software. To better capture the meaning of participants’ words, their quotes have been kept verbatim to retain the language style in which they shared (despite differences from standard English grammar). One member (DC) of our team compared the audio and transcripts to verify accuracy for all transcripts. For the focus group and those interviews that were conducted in the local Indigenous language, translation was carried out by two members of our team. The initial Indigenous language translation to English was carried out by one team member (DJE), with a second member (DC) going through the transcripts to ensure completeness and to see which words needed further clarification. An advanced Indigenous language speaker was then consulted to clarify any words in the transcripts where a deeper meaning might need further translation support. Interview and focus group transcripts were de-identified and stored on a secure server by a member of our team. An internal master list was created, linking participant study IDs with quotes. Team members also checked directly with some participants to confirm the English interpretation of their quotes.

After the interviews and focus group discussion were transcribed, they were then uploaded to NVivo software (version 14.24.3(7)) for qualitative data analysis. Reflexive thematic analysis (TA) using the method outlined by Braun and Clark [38] was carried out to support the characterization of key themes from the data. The first author (DC) carried out preliminary inductive coding of all the transcripts. The senior author (NR) was brought in for auditing, as well as for the refinement of the codes towards themes. Reflexive TA was used as it recognizes that themes provide an interpretive story of the data, are active, and are characterized from the community-based data [38]. Additionally, reflexive TA respected the specific community’s epistemology and ethics, including through reflective engagement with the data and promoting collaborative and reciprocal spaces among our team and with the participants.

## 3. Results

Ten semi-structured interviews and one focus group (*n* = 4) were conducted between December 2024 and January 2025. All participants were Indigenous and resided on a rural reservation in the southwestern USA. Six females and four males participated in the interviews, with an age range of 38 to 89 years of age. The mean age for the interviews was 70 years of age. Two females and two males participated in the focus group. The mean age of the focus group was 64 years old. For the focus group, questions were initially asked in English; however, participants chose to respond entirely in the local Indigenous language, which was respected. The findings from the interviews and focus group are presented in detail below. Through reflexive thematic analysis we characterized six themes from the interview data, summarized below in Table 1 and detailed further below in the text. Reflexive thematic analysis was also used to characterize four themes from the focus group, with data summarized at the end of this section.

**Findings from the Interviews.**

**Table 1:** Themes and subthemes from semi-structured interviews.

Theme	Subtheme
Historical and current barriers impact the sharing of TEK among our People	
Our TEK reveals changes to our Lands over time	
Preserving our language is necessary for intergenerational transmission of our TEK	-Sharing our TEK is needed in more community spaces -There is an urgency to learn traditional knowledge from Elders
Protecting our Lands and medicines is vital to our health and well-being	-Protecting Land through TEK
We must take the time to learn our TEK for future generations	-Importance of cultural protocols -TEK is collectively grounded in spirituality and promotes community wellness
We need to protect our TEK just as our ancestors did	-Community-level capacity and infrastructure challenges for physical repository protection

### 3.1. Historical and Current Barriers Impact the Sharing of TEK Among Our People

Throughout the interviews, there was discussion on the different barriers and factors that have impacted the sharing of TEK among local communities. Some participants described how intergenerational sharing of TEK continues to be affected by colonization, including the introduction of the boarding school system. An Elder male participant shared,

*[T]he main teachers were…the Elders, the Grandma and Grandpa. They* [the government] *took them away from them. Put them in school. That’s where we lost; that’s what was planned to begin with, 150 years ago*.

(US010)

These colonial events were seen to have deep and everlasting impacts on past and current generations. One participant expressed,

*[T]he barriers kind of goes all the way back to our historical trauma and…what happened at boarding schools and what was done there to our people to rid them of the language, their traditional knowledge, their family conception, things like that*.

(US009)

A decline in Indigenous language fluency was also identified among participants as a barrier for TEK transmission. Other language-related barriers that participants shared included the willingness to learn among younger generations being affected by other knowledgeable individuals. A male participant asserted that, “*…the hardest barrier is…when you have, you know, auntie, uncle, grandma, grandpa who’s like, no you don’t say it like that*” (US004). The need for more safe teaching approaches that are perceived by language learners as being less harsh or judgmental was identified as key to alleviating this barrier between older and younger generations.

Current barriers affecting the sharing of TEK were noted, including technology. One Elder participant shared in regard to the younger generation:

*Like for instance, computers. It is something not from us…but they are happy and learning from it. Even though it may be good, [but] in a way it is hurting…our children*.

(US005)

Technology was perceived as a distraction among younger generations that may inhibit their interest in learning their Indigenous language and TEK. Current social issues such as substance abuse were also noted as barriers that can limit the potential of younger generations to learn their TEK. Another Elder participant stated,

*So now it is “no, do not drink, do not run around at night” is what would be said. Now, no one listens to that anymore. All you hear is “I know”*.

(US006)

Other Elder participants shared the view that they want to teach younger people in their community, but there is a lack of listening.

*If they really want to know the old ways, you know, they need to sit down and listen to you.*

(US001)

Overall, participants discussed various barriers, including those that may hinder and/or prevent intergenerational transmission and learning of TEK.

### 3.2. Our TEK Reveals Changes to Our Lands over Time

During the interviews, participants shared their observations of how the Land and climate have changed over time and how this can affect the protection of TEK in the community. Since a majority of the participants were Elders, they provided reflections on changes over a longer period of time than younger participants. One Elder shared,

*Well, look at the weather now. It’s what, December. We used to have about a foot or two feet of snow already. It’s still summer here*.

(US001)

There was a shared concern among participants on ongoing drought conditions. Some participants expressed their concern over how this could affect the broader community and ceremonial settings. One participant stated that,

*Elders said years ago, this was never an issue. It was never a problem. You look at the river, it’s high. We’re good. Just keep going. But now it’s like, what are we going to do when this river dries up*.

(US004)

There was some discussion among participants on the changes in the Land over time and how initial place-based Indigenous names pinpoint changes in those very places. For example, a place may have been named based on certain vegetation in the area, but now that place no longer has that vegetation. These changes were noted and observed by some participants. One participant noted,

*Those are indications that those areas used to contain a lot of water, and now they don’t. So with…climate change, just our very place names that were in* [Indigenous language] *could tell you already that there has been an effect on the land based on how they used to name…the land*.

(US009)

The intimate connection between place-based names and the TEK of the Land was shared during interviews with some of the participants. The interviews provided further insight into how local lands are changing due to different climate and weather patterns in the region.

### 3.3. Preserving Our Language Is Necessary for Intergenerational Transmission of Our TEK

The intergenerational transmission of TEK was discussed by many participants during the interviews as being vital to knowledge protection. Several participants expressed how important it is that these knowledges are not lost. An Elder asserted that *“[i]t’s better to teach the young ones what you know. Pass it on. Don’t keep it to yourself”* (US001). The shared community responsibility of teaching and learning TEK was discussed by all participants. For example, one Elder stated that, *“[i]f you belong to a clan that has a knowledge of doing certain things, you know...That’s your responsibility”* (US003) to pass that knowledge on. The Elder provided an example of a younger male in his community who overcame language barriers to ensure ceremonial knowledge was not lost.

*So that was one barrier that he ran into because he didn’t know how to speak the language, but he learned it*.

(US003)

The importance of Indigenous language preservation and its significant role in learning and protecting TEK was discussed by several participants. Furthermore, it was noted how “sharing our TEK is needed in more community spaces” (*subtheme*) including through the language Some participants shared how they have seen dire changes in language fluency in their community over time. One Elder stated that,

*When I was growing up, I had always heard the* [Indigenous language] *being spoken to me, and I did not understand English when I was growing up*.

(US005)

Some participants noted that today the Indigenous language is not as often the first language in a home. One Elder stated that, *“[t]oday, the people…the children…all speak English. Even when they are little, they speak English already”* (US006). The gradual decline in fluent Indigenous language speakers was seen as concerning for TEK, as TEK is seen as being directly embedded in the language itself. One participant stated,

*Preserve the language because everything is tied to it*.

(US004)

Many participants articulated how crucial the language is to understanding TEK. One Elder shared:

*So, if you’re trying to pass on the knowledge like that and you don’t speak the language and stuff, then you can’t learn it because you can’t understand it*.

(US003)

Preserving the language was seen by participants as necessary to protecting their community’s collective knowledge systems, including TEK.

The roles of both Elders and younger people were also seen as vital to TEK transmission among participants. Nevertheless, while intergenerational transmission was broadly seen as important by participants, there were different viewpoints on how that could be achieved. One participant shared their approach of being open to teaching.

*I always tell them I don’t know everything, but this is what little I know from my grandparents, you know. And I hope you listen to me and I hope you…learn it…You won’t be ridiculed. You know, you’re here to learn. If you want to learn, that’s all you need*.

(US004)

Many participants additionally noted that the traditional ways of sharing knowledge have evolved, with some noting observed shifts in the home environment. The participants also expressed the value of home- and community-based TEK teachings that can be applied throughout life, including outside settings (i.e., off the Tribal reservation).

*So, we all gather at the* [traditional home structure]*, and that’s where Grandma and Grandpa, you know, and, um, they tell you stories that they go, you go by and…now I guess it’s when they have* [ceremony] *where they socialize, but not as…important like in the past*.

(US008)

It was also shared by several participants that “there is an urgency to learn traditional knowledge from Elders” (subtheme) and that this was foundational to sharing knowledge.

Participants also voiced how critical it is that those with deep knowledge share with those they trust to steward TEK into the future. One Elder shared a brief example from his community of the impacts of not carrying out this process of knowledge transmission to future generations.

*And she passed away, and it was never passed on to anybody else. So when she passed away, it stopped there*.

(US003)

### 3.4. Protecting Our Lands and Medicines Is Vital to Our Health and Well-Being

Throughout the interviews there were repeated notes among participants that to protect their community’s knowledges their People must also continue to protect their Lands and medicines. Many Elder participants noted the importance for current and future generations to protect and care for their Land. One participant shared that,

*Mother nature gives it to us free, for us to use…we can’t misuse our environments or…ecological stuff that was given to us to cure us, to help us*.

(US001)

The TEK-related cultural protocols and teachings around plants were seen to be a key part of this vital environmental protection role. For example, TEK-related teachings emphasize not overharvesting plants or selling them for monetary profit. Instilling this role and overall responsibility for Land and medicines among younger generations was also discussed by some participants. One participant shared their approach to passing on knowledge to young people:

*And…they have to understand that in the future, they’re the ones who are going to have to deal with a lot of the bad things that the past has…neglected. So today, we have to make a difference within yourselves and truly understand the importance of our land and the changes*.

(US009)

Many participants also shared how their communities have been taught through the generations about the importance of being in a reciprocal relationship with the Land.

*They say that if you take care of the land, the land will take care of you*.

(US008)

Some participants acknowledged that the role of TEK in their community has changed throughout time. “Protecting Land through TEK” (subtheme) was also expressed by some participants as a fundamental community teaching that has been impacted by past deliberate attempts to suppress their Peoples’ knowledges. One Elder stated,

*Everything changed…when we got invaded, the whole ecological system started to change. Our knowledge, our teaching[s], w[ere]n’t there anymore, and they put us in school*.

(US010)

Changes that followed colonization, including the establishment of the reservation system, were noted by some participants as harmful to the Indigenous stewardship of local Land.

### 3.5. We Must Take the Time to Learn Our TEK for Future Generations

Many participants shared a sense of urgency in protecting collective knowledges like TEK amid various concerns. They shared how ancestral knowledge is still valid, if not even more valuable, in guiding current and future generations. Participants reiterated how protecting TEK was also achieved by sharing, teaching, and learning. Several participants stated that foundational teachings, including those around land stewardship, were and should continue to be taught at home. Many of the participants shared valuable insights into this process. One Elder shared that “*[t]he foundation of that* [TEK transmission] *is the home. Compare that to the language it’s taught at home*” (US002). Several participants commented on the value of bringing teachings back to the home as their ancestors did. One Elder stated that,

*Everything has to start at home to where we teach our families, our relatives of how to protect our land, and many things that were taught to us by our Elders that were here before us*.

(US001)

There was also recognition among participants of how “TEK is collectively grounded in spirituality and promotes community wellness” (subtheme). Several participants made connections between knowledge and spirituality. Many of the Elder participants emphasized how non-denominational spirituality was widely valued and practiced when they were younger.

*There used to be a lot of praying people out there, and a lot of things will come out good*.

(US008)

Local Indigenous values (e.g., respect, reciprocity) that encompass knowledge were identified among some participants as central to protecting knowledge. An Elder shared:

*Believe in what you do, what you see, what you give, and what you respect. That’s…what knowledge is all about*.

(US001)

The “importance of cultural protocols” (subtheme) was discussed by some participants. Indigenous Natural Laws continue to guide Indigenous Peoples’ lifeways and conduct, including through cultural protocols. These Laws and their role in community structures were acknowledged by some participants. An Elder shared: “*I was born an* [Tribe name] *and I’m going to stick to that law…no matter what. I have respect for that*” (US008).

### 3.6. We Need to Protect Our TEK Just as Our Ancestors Did

Overall, all participants shared that their Peoples’ traditional knowledge was vital to cultural continuity and their way of life. Some participants also noted that they are cautious about who they share their knowledge with.

*And you can tell who really needs it. You can tell who wants it in a good way*.

(US010)

Many participants also saw the importance of protecting traditional knowledge and following cultural protocols on sharing different types of knowledge (e.g., gender-specific). Cultural protocols are key to guiding how, when, and whether different types of knowledge can be accessed by various individuals in Indigenous communities. Different knowledges may be appropriate only for individuals of certain ages, societies, and genders (e.g., women-specific knowledges) to access.

“Community-level capacity and infrastructural challenges for physical repository protection” (subtheme) was also discussed among some participants. Some noted that there is some value in placing culturally significant materials into physical repositories.

*I think that would be something…that should still be written down. And you take care of it. Put it in a fireproof case or something*.

(US001)

There were also concerns noted by participants that there are gaps in the capacity for community-level protection of current and future repositories. Furthermore, some participants saw the value in strengthening infrastructure to secure materials that contain cultural knowledge. Building a stronger repository to store and protect TEK-related materials was supported by some participants.

*Making a toolbox that would be for our people to have there always. Because, a lot of our people who have a lot of knowledge aren’t here anymore*.

(US009)

**Findings from the Focus Group.**

During the focus group with Elders, we found that similar themes were discussed as were presented in the interview findings (see Table 2). Despite these similarities, we present below some highlights that add to the voices presented from the interview findings and, therefore, equitably honor the voices of the Elders who participated in the focus group.

### 3.7. Stewardship of Our Land Is Our Responsibility

Participants in the focus group emphasized the importance of land stewardship for their communities. For example, some participants shared that it is paramount for younger generations to continue this land stewardship role in their communities.

*The Elderly back then would run things good. For us…recently it is like that, the ones that are coming after us, it would be good if they can preserve it*.

(US011)

All participants also shared their experiences in land protection through forest management and wildland firefighting and how important it is to continue this work into the future.

There was also discussion on how “community and family access to TEK” (subtheme) may have changed in local communities. Most of the participants reminisced similarly to the interviews on their upbringing and how knowledge was traditionally taught in their homes. Focus group participants equally echoed how important it is that foundational teachings are taught in family settings. They also shared on how “spirituality is vital to protecting our land” (subtheme) and adapting to changes in their community. An Elder stated: “*We do not know what we are going towards, so you have to pray*” (US014).

### 3.8. Teaching Language and TEK in Different Settings Is Key

During the focus group, as in the interviews, there was discussion among participants on the significance of Indigenous language preservation in their community.

There was also discussion on the need for stronger language and TEK-related learning opportunities, including in local schools. An Elder participant asserted: “*I think that the schools should teach [the]…*[Tribe name] *language in class and all of the traditional food and everything in class*” (US012).

### 3.9. Social Changes Can Hinder Our Younger Generations from Learning TEK

Focus group participants discussed various changes and concerns that they are currently observing in local communities. Concerns included youth engaging in harmful behaviors such as substance abuse. There was also discussion on how social “changes affecting knowledge uptake can lead to health concerns” (subtheme) and that this may be inhibiting younger generations from learning TEK and their Indigenous language. Participants reiterated the importance of intergenerational knowledge transmission, with one Elder stating:

*Like they are saying, we are handing it over to you, and now you need to carry it. Now it is like it was handed to us. It is good that we are talking about it*.

(US011)

### 3.10. Infrastructure and Physical Protection of TEK Concerns

While much discussion during the focus group encompassed protection of TEK through language and intergenerational transmission of knowledge, there was also some discussion, as in the interviews, on the need for enhanced physical protection of a current community repository. Some participants acknowledged that stronger protection of materials is needed, including the need to ensure the community building is protected from fire and other environmental threats. One Elder commented: “*Whatever that is there, we’re not going to get it again*” (US012). Most participants also felt it was important to enhance awareness of the need for these repository protections, including among community stakeholders and leadership. One Elder participant shared that, “*[i]f we do not notice* [the need for these protections]*, it* [a lack of prevention] *will catch up to us*” (US011).

## 4. Discussion

The findings from this study captured varied perspectives on how the Indigenous community wants to protect its TEK as well as areas for further development and needed support. We characterized six themes from the interview data, including the following: (1) Historical and current barriers impact the sharing of TEK among our People; (2) Preserving our language is necessary for intergenerational transmission of our TEK; (3) Our TEK reveals changes to our Lands over time; (4) Protecting our Lands and medicines is vital to our health and well-being; (5) We must take the time to learn our TEK for future generations; and (6) We need to protect our TEK just as our ancestors did. The themes identified in the focus group were very similar to the interview data, showcasing how individual and collective perspectives overlapped. Many of the participants in both the interviews and focus group were Elders, which was apparent by the significant knowledge shared on their experiences and observations of various concerns around TEK protection, community-level protections, potential gaps, and cultural continuity over the long term.

Many participants expressed that protection of TEK included the need for more intergenerational teaching and sharing among local communities. This study reinforced how significant the role of Elders is to the stewardship of Land, knowledge, and language preservation. Prioritizing and platforming TEK held by Elders was seen to be crucial for Indigenous health and well-being. This finding is similar to other research on the role of Elders in Indigenous communities. For example, research has shown that Elders are vital to the delivery and impact of successful Land-based programming for Indigenous youth in the USA and Canada [39]. Additionally, in a scoping review by Price et al. [39], they found that when Elders and other cultural experts transmit TEK to Indigenous youth through Land-based programs, it can support positive youth development and intergenerational connectedness and reinforce Land stewardship roles. By platforming the roles and leadership of Elders in strengths-based health programs in their communities, their involvement has been shown to be effective in promoting cultural continuity and community well-being. For example, the Qungasvik Project is a Yup’ik strengths-based project where Elders deliver activities to the community that promote culturally based protective factors among Yup’ik youth [40]. Community-based approaches that honor Elders and provide them with vast opportunities and spaces to teach younger generations TEK are urgently needed.

**Figure 1:**
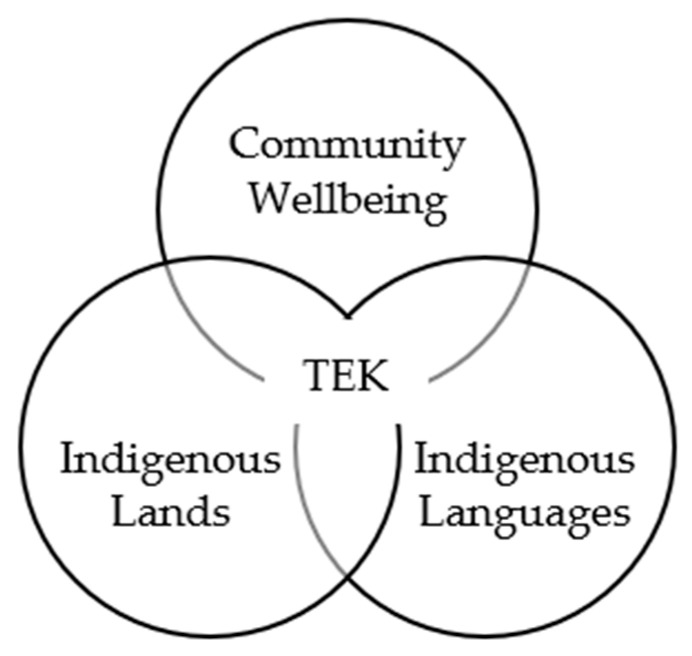
Protection of Indigenous Traditional Ecological Knowledges (TEK) is directly interconnected to Land, Indigenous Languages, and Community Well-being.

Although interview and focus group questions focused on community-level protection of TEK, the participants often spoke about the interconnectedness between TEK, Land stewardship, language preservation, and community well-being. This broadening of the context and interconnectedness of TEK to other topics differs from Euro-Western worldviews that may generally separate these areas into more siloed areas. The inseparability of the People, their Lands, and their knowledges was apparent in this study and overlapped with research in similar settings [41]. The protection of Indigenous Lands, Indigenous languages, and community well-being was seen as being connected with the overall data protection and the long-term vitality of TEK (see Figure 1).

This study therefore highlighted in essence the importance of strengthening Indigenous data sovereignty, with ‘data’ being seen as broadly including all forms of Indigenous TEK. As data are becoming increasingly digitized and more widely available, it is crucial that Indigenous Peoples can develop and/or strengthen data protection mechanisms, including repositories grounded in Indigenous rights and values [42]. When strong TEK data protection infrastructure is present in an Indigenous community, the data are more likely to be controlled and handled responsibly and ethically and will collectively benefit the community [42].

Findings from this study also exemplified the importance of TEK and its application to climate change. Indigenous stakeholders and environmental professionals recognize that the capacity of Indigenous Peoples to adapt to climate change is also threatened when their land bases shrink or change [43]. Supporting Indigenous Peoples to continue their roles as stewards of their Lands is imperative to the protection of their TEK as well as to overall planetary health and biodiversity integrity. There are examples of effective programming and policies that support Indigenous stewardship and climate change mitigation, including the Land Guardian (Canada) [44] and Ranger (Australia) [45] programs. Expansion of local Land protection programs, including in the southwestern USA, has the potential to promote community well-being and prepare Indigenous youth for these essential roles, as highlighted by the participants. Globally, there is a need for increased recognition of Indigenous Land rights and how Land rights are directly connected to TEK protection inclusive of Indigenous health (e.g., the protection of traditional medicines).

The preservation of Indigenous languages was highlighted in our study as being paramount to the protection of TEK. Indigenous languages are the heartbeat of Indigenous cultures, worldviews, and ways of life. Article 13 of the United Nations Declaration on the Rights of Indigenous Peoples (UNDRIP) recognizes the right of Indigenous Peoples to revitalize, use, and transmit their languages [46]. Indigenous language was platformed and honored at various times during this study, including in the research planning, engagement with key stakeholders, data collection, and data analysis. By carrying out research processes in the local Indigenous language, our research became embedded in the local epistemology and worldview. Indigenous languages are also instrumental in disseminating research findings back to the community, as was the case in this study. Indigenous languages are a direct vessel that connects biodiversity, TEK, and overall planetary stewardship, which was demonstrated by the wide conversation points broached by the participants that crossed these areas [15]. This study therefore aligns with other studies by highlighting that there continues to be a need to support culturally based protective factors in Indigenous communities, including Indigenous language programming, to prevent negative health outcomes for people and the planet [47,48,49]. Indigenous languages, for example, have been shown to have positive effects on Indigenous health, including protection against harmful behaviors [50] and the delivery of culturally safe healthcare [51].

Educational institutions were once a major source of Indigenous language degradation; however, some have evolved to be key sites of language revitalization [52]. There have been many exemplary Indigenous language school immersion programs, including the Hawaiian Language Medium Education schools and the Akwesasne Freedom School (AFS). Jimerson [53] describes how the language immersion approach at AFS challenges settler-colonial educational structures by platforming Haudenosaunee languages and worldviews. The school also contributes to the overall rebuilding of local healthy Indigenous communities, healing, and the reconnection to Land and culture [53]. Community-based Indigenous language programs can be particularly significant for engaging youth in promoting their identities and cultural confidence [50]. Indigenous language revitalization programs are the most successful when they are community-based [54]. However, the capacity of Indigenous communities to build a comprehensive language program may be limited. By reframing Indigenous language preservation and revitalization as health programming, there is much potential for Indigenous Peoples to leverage policy such as UNDRIP to support cultural continuity and TEK transmission in their communities. Our study identified the need for more robust programming that is aimed at increasing TEK transmission and language fluency, which may include the use of technology [54]. Finally, immersive Land-based language programs that strengthen intergenerational linkages have much potential [55] to also protect TEK.

Indigenous community well-being is dependent on various factors, including those highlighted in this study. Various resources have been invested in intergenerational programming in the context specific to this study, including cultural educators in schools and institution-based programs. Our study highlights, however, the need for more comprehensive approaches to supporting community-based initiatives that protect TEK and Indigenous languages. Community-based and -led efforts that are embedded in community knowledge and do not rely heavily on outside funding can promote more sustainable intergenerational programming. Supporting Indigenous communities to protect their TEK also enables them to build a stronger collective future grounded in self-determination, cultural strengths, Indigenous knowledges, and connectedness.

Overall, our participants highlight the importance of not viewing TEK in a vacuum but through a holistic, interconnected, systems-based approach that sees it as being interconnected to healthy Lands, intergenerational knowledge transmission, Indigenous languages, and the safe and sustainable storage of Indigenous data for generations to come. Linear Euro-Western-centric approaches to research will likely miss out on the systems perspective inherent in Indigenous worldviews that will be required for understanding all of the elements important in the overall protection of TEK (i.e., the past, context elements, where we are now, and where we might go into the future). Future research into TEK protection could focus on developing practical guidance or toolkits for communities on how to move to operationalize TEK data storage in the digital age while considering Indigenous data sovereignty principles and practices relevant to a respective Indigenous community.

### Limitations and Strengths

This qualitative study was carried out in a southwestern rural Indigenous community in the USA with its own specific knowledge system, so its transferability to other contexts may therefore be limited. Given similar findings to other research studies on TEK protection and the shared experience of colonization, however, there are likely similarities to consider. Regardless, the findings from this research study may not be representative of all Indigenous Peoples, including other American Indian Peoples, whose knowledges and experiences are unique to their communities. Although this study included community perspectives, it also may not be representative of the entire community’s viewpoints on the topics addressed, including across ages, genders, and geographic areas (e.g., there are members from this Tribal Nation that currently reside in urban areas). Regardless, given the similarities in the perspectives shared in the interviews and the focus group, we feel the findings are useful for community planning purposes locally and regionally. Additionally, as a considerable amount of the project was conducted in the local Indigenous language, we acknowledge that some of the meaning may not have been fully captured in English. Although we did our best to translate the Indigenous words from participants’ quotes to English, there may be some cultural nuances from the verb-based Indigenous language that have been lost, and the translation may not encompass the true meaning of the words. Nevertheless, we have made substantial efforts to ensure that the embodied meanings shared by participants were captured. This process was strengthened by having bilingual co-authors fluent in both the Indigenous language and English.

## 5. Conclusions

This Indigenous-led study attempted to honor the knowledges, perspectives, and experiences of the participants and the communities they come from. Participants in this study provided valuable insight into how an Indigenous community seeks to protect and steward its TEK, as well as what is needed to ensure community-level TEK protection into the future. Cultural protocols that guide knowledge protection and stewardship were also explored and discussed by participants. This study exemplifies the need for further support of Indigenous communities’ capacity to protect their knowledges and strengthen their overall TEK stewardship. The importance of TEK stewardship was additionally found to be related to the health and well-being of the Land and people. The preservation and revitalization of Indigenous languages was also strongly highlighted as being necessary for the intergenerational knowledge transfer of TEK. Overall, vibrant and healthy Indigenous Lands and languages are necessary for optimal community well-being and health, as well as Indigenous knowledges stewardship. It is imperative that Indigenous communities, including the one in this study, are able to enhance and ensure intergenerational transmission of their knowledges including TEK. The health of Indigenous Peoples is a recognized determinant of planetary health [15]. The stewardship and protection of TEK therefore continues to be significant to the health and well-being of current and future generations of Indigenous Peoples on Turtle Island, as well as all beyond.

## Figures and Tables

**Table 2:** Themes and subthemes from the focus group.

Theme	Subtheme
Stewardship of our Land is our responsibility	-Community and family access to TEK is key -Spirituality is vital to protecting our land
Teaching language and TEK in different settings is key	
Social changes can hinder our younger generations from learning TEK	-Changes affecting knowledge uptake can lead to health concerns
Infrastructure and physical protection of TEK are concerns	

## Data Availability

Due to Indigenous ethical considerations and Indigenous data sovereignty, any data and materials associated with this research will not be available unless additional relevant Indigenous ethics agreements are in place with the local community. Further inquiries can be directed to the corresponding author.

## Supplementary Materials

The following supporting information can be downloaded at: https://www.mdpi.com/article/10.3390/ijerph22101573/s1, File S1: Interview and focus group guides and File S2: Codebooks.
