# Supplementary material for: Knowledge Connects Our Hearts and Lands: A Qualitative Research Study on Stewarding Indigenous Traditional Ecological Knowledges for Community Well-Being
Source: Int J Environ Res Public Health. 2025 Oct 15;22(10):1573. doi: 10.3390/ijerph22101573 (PMC12562921; doi:10.3390/ijerph22101573)
Supplement: Supplementary file 1 [file ijerph-22-01573-s001.zip › ijerph-3869833-supplementary/S1-Interview codebook S1 Qual res study Indigenous TEK.pdf]

Knowledge connects our hearts and Lands: A qualitative research study on stewarding Indigenous traditional ecological knowledges for community wellbeing

Name	Description
**Historical and current barriers impact the sharing of TEK among our People.**	Various barriers were discussed by participants including those stemming from historical trauma and colonization. Religion was also highlighted as a barrier that can hinder and inhibit the learning and sharing of traditional ecological knowledge (TEK).
**Preserving our language is necessary to intergenerational transmission of our TEK.**	Intergenerational transmission of TEK including through language preservation was discussed by participants as being crucial.
Urgency to learn traditional knowledge from Elders	Various examples were given by participants showing the urgent need to listen to and learn from Elders still around the community. They noted that many of those they knew and learned from have passed on.
Sharing our TEK is needed in more community spaces	Preserving TEK and the local Indigenous language emerged as a key theme among participants. There was a direct connection made between protecting TEK and protecting the Indigenous language that it is contained within.
**Our TEK reveals changes to our Lands over time.**	Various aspects of climate change were discussed by participants including their observation of environmental changes such as drought. They noted there has been changes in weather throughout their lifetimes.
**Protecting our Lands and medicines is vital to our health & wellbeing.**	Traditional medicine teachings were touched upon by various participants including values important to protecting the land and

Name	Description
	plants. The roles of those with traditional medicine knowledge was also highlighted.
Protecting Land through TEK	Participants discussed aspects of land protection through their knowledge.
**We must take the time to learn TEK for future generations.**	Foundational teachings surrounding TEK were shared by participants including the importance of learning at home. Participants also highlighted the need for younger generations to embrace learning and listening as it was done in the past.
Importance of cultural protocols	Cultural protocols surrounding TEK were discussed by participants including gender knowledges and why protocols still need to be followed and taught.
TEK is collectively grounded in spirituality and promotes community wellness	Spiritual aspects of learning and respecting TEK were discussed including the importance of prayer and connection to the Creator. TEK was also described as significant to promoting overall community wellness and harmony.
**We need to protect our TEK just as our ancestors did.**	Protecting traditional knowledge was discussed as significant by participants. They expressed the need to protect the land that TEK comes from. The need to strengthen community infrastructure and capacity to protect, store, and provide access to TEK was also highlighted.
Community-level capacity and infrastructure challenges	Different aspects of a lack of capacity and infrastructure were discussed by participants. They shared concern for the need for better storage and protection of TK.
