# Supplementary material for: Knowledge Connects Our Hearts and Lands: A Qualitative Research Study on Stewarding Indigenous Traditional Ecological Knowledges for Community Well-Being
Source: Int J Environ Res Public Health. 2025 Oct 15;22(10):1573. doi: 10.3390/ijerph22101573 (PMC12562921; doi:10.3390/ijerph22101573)
Supplement: Supplementary file 1 [file ijerph-22-01573-s001.zip › ijerph-3869833-supplementary/S2-Focus group Codebook S1 Qual res study Indigenous TEK.pdf]

Knowledge connects our hearts and Lands: A qualitative research study on stewarding Indigenous traditional ecological knowledges for community wellbeing

Name	Description
**Infrastructure & physical protection of TEK concerns**	Participants spoke about the need to strengthen and develop further infrastructure to secure and protect culturally significant materials. They particularly identified building more fireproof repository buildings to protect these materials.
**Social changes can hinder our younger generations from learning TEK**	Participants discussed concern on social changes that are affecting their communities especially younger generations. These concerns included substance abuse and drugs. These issues can be barriers to intergenerational transmission of TEK and cultural continuity.
Changes in knowledge can lead to health concerns	Some examples were provided by participants on how a lack of knowledge (i.e., traditional foods) can impact health.
**Stewardship of our Land is our responsibility**	All participants spoke about their experiences of stewardship including wildland fire fighting. They shared concern about changes in weather and land including prolonged drought conditions.
Community and family access to TEK	The varying levels of access to TEK among the local community and families was discussed.
Spirituality is vital to protecting our land	Spirituality was discussed broadly as being important to protecting land. Some observed changes in spirituality were touched upon.

Name	Description
**Teaching language & TEK in different settings is key**	Participants spoke about how teaching the Indigenous language is the foundation to being able to learn and understand TEK in different contexts.
